# Horizontal gene transfer from *Agrobacterium* to plants

**DOI:** 10.3389/fpls.2014.00326

**Published:** 2014-08-11

**Authors:** Tatiana V. Matveeva, Ludmila A. Lutova

**Affiliations:** Department of Genetics and Biotechnology, St. Petersburg State UniversitySt. Petersburg, Russia

**Keywords:** *Agrobacterium*, T-DNA, horizontal gene transfer, *Nicotiana*, *Linaria*

## Abstract

Most genetic engineering of plants uses *Agrobacterium* mediated transformation to introduce novel gene content. In nature, insertion of T-DNA in the plant genome and its subsequent transfer via sexual reproduction has been shown in several species in the genera *Nicotiana* and *Linaria*. In these natural examples of horizontal gene transfer from *Agrobacterium* to plants, the T-DNA donor is assumed to be a mikimopine strain of *A. rhizogenes*. A sequence homologous to the T-DNA of the Ri plasmid of *Agrobacterium rhizogenes* was found in the genome of untransformed *Nicotiana glauca* about 30 years ago, and was named “cellular T-DNA” (cT-DNA). It represents an imperfect inverted repeat and contains homologs of several T-DNA oncogenes (Ng*rolB*, Ng*rol*C, NgORF13, NgORF14) and an opine synthesis gene (Ng*mis)*. A similar cT-DNA has also been found in other species of the genus *Nicotiana*. These presumably ancient homologs of T-DNA genes are still expressed, indicating that they may play a role in the evolution of these plants. Recently T-DNA has been detected and characterized in *Linaria vulgaris* and *L. dalmatica*. In *Linaria vulgaris* the cT-DNA is present in two copies and organized as a tandem imperfect direct repeat, containing Lv*ORF2*, Lv*ORF3*, Lv*ORF8*, Lv*rolA*, Lv*rolB*, Lv*rolC*, Lv*ORF13*, Lv*ORF14*, and the Lv*mis* genes. All *L. vulgaris* and *L. dalmatica* plants screened contained the same T-DNA oncogenes and the *mis* gene. Evidence suggests that there were several independent T-DNA integration events into the genomes of these plant genera. We speculate that ancient plants transformed by *A. rhizogenes* might have acquired a selective advantage in competition with the parental species. Thus, the events of T-DNA insertion in the plant genome might have affected their evolution, resulting in the creation of new plant species. In this review we focus on the structure and functions of cT-DNA in *Linaria* and *Nicotiana* and discuss their possible evolutionary role.

## Introduction

Horizontal gene transfer (HGT) takes place widely in prokaryotes, where its ecological and evolutionary effects are well-studied (Koonin et al., 2001). Comparative and phylogenetic analyses of eukaryotic genomes show that considerable numbers of genes have been acquired by HGT. Gene acquisition by HGT is therefore a potential creative force in both eukaryotic and prokaryotic genome evolution. However, mechanisms of HGT are poorly understood in the Eukaryota in comparison to gene transfer among the Procaryotae. The persistence of horizontally transferred genes in some organisms may confer selective advantages (Koonin et al., 2001; Richardson and Palmer, 2007). Most examples of HGT in higher plants involve the transfer of chloroplast or mitochondrial DNA and have been the subject of numerous reviews (Dong et al., 1998; Richardson and Palmer, 2007). There are few descriptions of horizontal transfer of nuclear genes between species. One example is transfer of the gene that codes for the cytosolic enzyme phosphoglucose isomerase predicted to have occurred between *Festuca ovina* and some species from the genus *Poa* (Ghatnekar et al., 2006; Vallenback et al., 2008, 2010). Evidence of gene transfer from bacteria to the nuclei of multi-cellular eukaryotes is rare (Richards et al., 2006; Acuna et al., 2012). HGT from bacteria to plants has been restricted to *Agrobacterium rhizogenes* and representatives of genera *Nicotiana* and *Linaria*, and represents some of the most recent transfers in evolution (White et al., 1983; Intrieri and Buiatti, 2001; Matveeva et al., 2012; Pavlova et al., 2013).

*A. rhizogenes*, and the related bacterium *A. tumefaciens*, transform a wide variety of host plants by transferring a segment of the large tumor-inducing plasmid, called T-DNA, into host cells (White et al., 1982; Otten et al., 1992; Veena et al., 2003; Tzfira and Citovsky, 2006; Vain, 2007). The T-DNA is integrated through non-homologous recombination into the host cell genome where it is expressed. Expression of T-DNA genes results in the formation of hairy roots or crown galls, that are transgenic tissues, formed on a non-transgenic plant. This phenomenon is called “genetic colonization,” one of the examples of the host-parasite relationship (Tzfira and Citovsky, 2006). It is unclear whether or not colonized plants have received benefits from such colonization, however, we could expect that it is beneficial in some cases since there are footprints of HGT from *Agrobacterium* to plants in the genomes of several present day plant species.

## T-DNA in *Nicotiana glauca*

In early investigations of *Agrobacterium* mediated transformation of plants, most researchers assumed that there was no significant homology to the T-DNA in untransformed plant genomes. White et al. (1982) attempted to detect pRiA4b T-DNA sequences in the genome of *Nicotiana glauca*, transformed in laboratory conditions by *Agrobacterium rhizogenes* strain A4. Southern analysis detected a fragment of pRiA4 in the transgenic tissue. Surprisingly, a hybridization signal was also detected in uninfected tissues of *N. glauca*. Further analysis confirmed the presence of DNA homologous to T-DNA in the *N. glauca* genome. This homologous DNA was referred to as “cellular T-DNA” (cT-DNA) (White et al., 1983).

Furner et al. (1986) investigated *Nicotiana glauca* plants, collected in geographically separated territories. Southern analyses showed the presence of cT-DNA in all studied varieties of *N. glauca*. Sequencing of the *N. glauca* cT-DNA demonstrated that it was organized as an imperfect inverted repeat. The left arm of cT-DNA, containing *rolB* and *rolC* homologs (Ng*rolB* and Ng*rolC*L) was more extended than the right arm, which contained only the *rolC* homolog (Ng*rolC*R). The coding sequences of Ng*rolB* and Ng*rolC*R were found to contain early stop codons.

Subsequent analysis of the nucleotide sequence of this cT-DNA identified open reading frame 13 (ORF13) and ORF14 homologs in both the left and right arms, called NgORF13L, NgORF14L, NgORF13R, and NgORF14R, respectively (Aoki et al., 1994).

**Figure 1:**
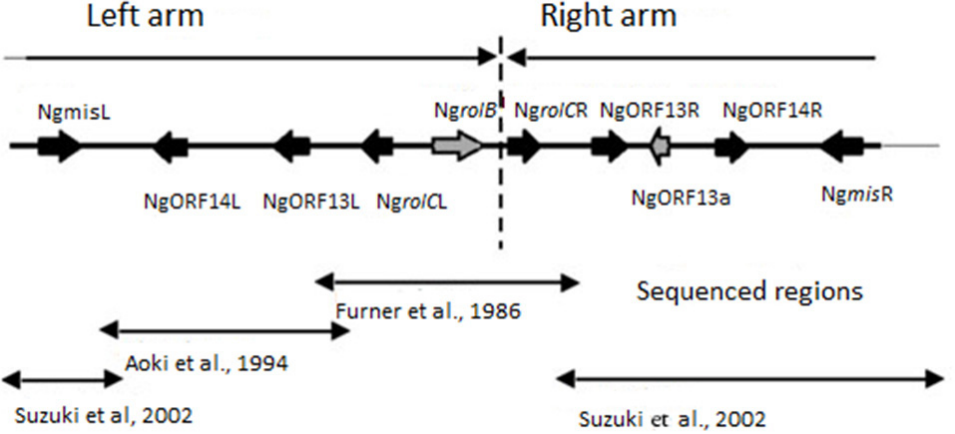
**Structure of cT-DNA in the *Nicotiana glauca* genome (based on Suzuki et al., 2002)**. The cT-DNA and its flanking regions are indicated. Lines with a single arrowhead indicate the imperfect inverted repeat. Lines with arrowheads at both ends indicate regions sequenced by each of three groups.

In 2001 Suzuki et al. characterized *A. rhizogenes* strain MAFF301724 and described a new opine synthase gene (mikimopine synthase gene *mis*). A part of the *mis* gene displayed strong homology to distal fragments of *N. glauca* cT-DNA, called Ng*mis*L and Ng*mis*R, respectively (Suzuki et al., 2002). Suzuki et al. (2002) suggested that the complete cT-DNA region of *N.glauca* is comprised of the 7968 bp left arm and 5778 bp right arm that were derived from the T-DNA of a mikimopine Ri plasmid similar to pRi1724. The level of nucleotide sequence similarity between the left and right arms is greater than 96% and the gene order is conserved suggesting a duplication event. The structure of the *N.glauca* cT-DNA is summarized in Figure 1. Since cT-DNA has been identified in all studied varieties of *N. glauca* (Furner et al., 1986), it is reasonable to suggest that the transformation event occurred before the formation of this species. This suggests that other related species may contain cT-DNA.

## T-DNA in other *Nicotiana species*

The genus *Nicotiana* is one of the largest genera in the Solanaceae and contains 75 species that are characterized by a wide range of variations among their floral and vegetative morphology (Clarkson et al., 2004). The different *Nicotiana* species evidence interspecific crosses which complicates *Nicotiana* phylogeny. Goodspeed hypothesized that there are two distinct lineages in *Nicotiana* which arose from two ancestral pre-petunioid and pre-cestroid lineages. He supposed that the base chromosome number of the genus was 12 and stressed the role of doubling and hybridization in *Nicotiana* evolution. Goodspeed divided *Nicotiana* into three sub-genera *Rustica, Tabacum*, and *Petunioides* and 14 sections (Goodspeed, 1954). Since then, the number of subgenera of *Nicotiana* has remained constant, while the number and composition of the sections has been revised (Clarkson et al., 2004). 75% of tobacco species originate from the Americas and 25% of species are from Australia (Goodspeed, 1954; Clarkson et al., 2004).

Identification of T-DNA in *N. glauca* raises two questions: what other *Nicotiana* species contain cT-DNA, and what was the pattern of dissemination within the group?

To answer the first question Furner et al. (1986) examined the genomes of 17 species of the genus *Nicotiana*. Using Southern analyses he showed that only six species from the subgenera *Rustica* and *Tabacum* contained sequences homologous to the *rol* genes of *Agrobacterium rhizogenes*. These species are *N. glauca*, *N. otophora, N. tomentosiformis, N. tomentosa, N. benavidesii, N. tabacum*. Examination of T-DNA- like sequences in *N. tabacum* has shown that it contains a *rolC* homolog and two ORF13 homologs (t*rolC*, tORF13-1 *and* tORF13-2, respectively) (Meyer et al., 1995; Frundt et al., 1998). Intrieri and Buiatti studied the distribution and evolution of *Agrobacterium rhizogenes* genes in the genus *Nicotiana.* Forty two species representing all *Nicotiana* sections were examined for the presence of *rolB*, *rolC*, ORF13, and ORF14 homologs in their genomes. T-DNA-like sequences detected were compared with each other and with contemporary sequences of *Agrobacterium*. The results demonstrated the presence of at least one T-DNA gene in each of 15 *Nicotiana* species representing all three subgenera. All currently available data on the distribution of cT-DNA among *Nicotiana* species are summarized in Table 1.

**Table 1:** **Distribution of T-DNA-like sequences among *Nicotiana* species**.

	Section^*^	**Species**	**T-DNA genes**	**Sequence Acc#**	**References**
*Rustica*	*Paniculatae*	N.glauca^*^	*+(rolB-mis)*	X03432.1; D16559.1 AB071334.1; AB071335.1	1, 2, 3, 4^**^
		*N.paniculata*	*–*		2, 5
		*N.knightiana*	*–*		2, 5
		*N.solanifolia*	*–*		5
		*N.benavidesii*	*+(rolC)*	n/a^***^	2, 5
		*N.cordifolia*	*+(rolB-ORF14)*	AF281252.1 AF281248.1 AF281244.1	2, 5
		*N.raimondi*	*–*		5
	*Rusticae*	*N.rustica*	*–*		2, 5
*Tabacum*	*Tomentosae*	*N.tomentosa*	*+(ORF13-mis)*	n/a	2, 4
		*N.tomentosiformis*	*+(rolC-mis)*	AF281249.1 AF281245.1 AF281241.1	2, 4,5
		*N.otophora*	*+(rolC-ORF14)*	AF281250.1 AF281247.1 AF281243.1	2, 5
		*N.setchelli*	*+(rolC)*	n/a	2
	*Nicotiana*	*N.tabacum*	*+(rolC-mis)*	AF281246.1 AF281242.1	2,4,5
*Petunioides*	*Undulatae*	*N.glutinosa*	*–*		2, 5
		*N.undulata*	*–*		5
		*N.arentsii*	*+(rolC)*	n/a	5
	*Trigonophyllae*	*N.trigonophylla*	*–*		5
	*Sylvestris*	*N.sylvestris*	*–*		5
	*Alatae*	*N.langsdorffi*	*–*		2, 5
		*N.alata*	*–*		5
		*N.longiflora*	*–*		5
		*N.forgetiana*	*–*		5
		*N.sanderae*	*–*		5
		*N.plumbaginifolia*	*–*		5
	*Repandae*	*N.nesophila*	*–*		5
		*N.stocktonii*	*–*		5
		*N.repanda*	*–*		5
		*N.nudicaulis*	*–*		5
	*Noctiflorae*	*N.noctiflora*	*–*		5
		*N.petunioides*	*–*		5
	*Petunioides*	*N.acuminata*	*(rolC)*	n/a	5
		*N.pauciflora*	*–*		5
		*N.attenuata*	*–*		5
		*N.miersii*	*+(rolB)*	n/a	5
	*Bigelovianae*	*N.bigelovi*	*+(rolB)*	n/a	5
	*Polydiclae*	*N.clevelandi*	*–*		5
	*Suaveolentes*	*N.umbratica*	*–*		5
		*N.debneyi*	*+(rolC)*	AF281251.1	5
		*N.gossei*	*+(rolC)*	n/a	5
		*N.rotundifolia*	*–*		5
		*N.suaveolens*	*+(rolC)*	n/a	5
		*N.exigua*	*+(rolC)*	n/a	5
		*N.goodspeedii*	*–*		5

* Nicotiana sections from Knapp et al. (2004) and N.glauca section is from Goodspeed (1954);

** 1, White et al., 1983; 2, Furner et al., 1986; 3, Aoki et al., 1994; 4, Suzuki et al., 2002; 5, Intrieri and Buiatti, 2001;

*** n/a, not available.

It is important to note, that there are some inconsistencies among the data by Furner et al. (1986) and Intrieri and Buiatti (2001). For example, Intrieri and Buiatti (2001) showed that T-DNA is present in *N. debneyi* and *N. cordifolia*. Furner et al. (1986) found no T-DNA in these species. This contradiction requires additional studies.

Thus, to date, T-DNA was found in every *Nicotiana* subgenus which include species, native to America and Australia (Goodspeed, 1954).

Phylogenetic analyses were performed by Intrieri and Buiatti (2001) to compare nucleotide sequences of cT-DNA in several *Nicotiana* species with the T-DNA of *Agrobacterium*. The following species were used in the analyses and represented all three subgenera: *N. cordifolia* (subgenus *Rustica* sec. *Paniculatae*); *N. tomentosiformis* and *N. otophora* (subgenus *Tabacum*), *N. tomentosiformis* (participated in *N. tabacum* speciation together with *N. sylvestris*); *N. glauca* used to be included in the subgenus *Rustica* sec. *Paniculatae* (Goodspeed, 1954), but later it was moved to the sec. *Noctiflorae* of the subgenus *Petunioides* (Knapp et al., 2004); and *N. debneyi* [*Suaveolentes*, an Australian section of *Nicotiana*, and a polyploid species of the subgenus *Petunioides* (Knapp et al., 2004)].

Analysis of nucleotide sequences revealed that *N. cordifolia* and *N. glauca rolB, rolC*, ORF13, and ORF14 genes show a high level of sequence similarity (93.5–98.5%). These data indicate that *N. cordifolia* and *N. glauca* are related species and are consistent with the proposal of Goodspeed (1954) that both species should be included in subgenus *Rustica* sec. *Paniculatae*. Similar clustering was found between the representatives of the subgenus *Tabacum*. Sequence similarities were lower between *Rustica* and *Tabacum* species, ranging from 66.3 to 68.6% for *rolC* and from 70.2 to 82.9% for ORF13 but was higher for ORF14 (94–97%). Surprisingly, the petunioid *N. debneyi rolC* gene demonstrates high sequence similarity (93.4%) with the *N. glauca rolC* gene, but lower similarity (around 67%) with those found in species belonging to the subgenus *Tabacum*. It was speculated that the polyploid species *N. debneyi* got cT-DNA from an ancestor of sec. *Paniculatae*. The homologies suggest that the *Nicotiana rol* genes parallel *Nicotiana spp*. evolution, being divided into two clusters, one that includes *N. glauca, N. cordifolia*, and *N. debneyi*, the second comprising species from the subgenus *Tabacum* (Intrieri and Buiatti, 2001).

Present day cT-DNA genes clustered with each other, making it difficult to predict which Ri-plasmid would be the source of the cT-DNA in each *Nicotiana* species. Since the pace of evolution differs between bacteria and plants, and since the pRi T-DNAs may have undergone rearrangements with each other (Moriguchi et al., 2001), it is difficult to define which ancient T-DNA was the origin of cT-DNA in different *Nicotiana* species using such phylogenetic analysis (Tanaka, 2008).

Another option for exploring the origin of cT-DNA is opine typing which was performed by Suzuki et al. (2002). They identified opine gene homologs in *N. glauca* (Ng*misL and* Ng*misR*, respectively) and screened 12 *Nicotiana* species for *mis* homologs using Southern blot hybridization. The analyses included five species from the subgenus *Rustica* (*N. glauca, N. benavidesii, N. paniculata, N. knightiana, N. rustica*), five species from the subgenus *Tabacum* (*N. tomentosa, N. tomentosiformis, N. otophora, N. tabacum and N. glutinosa*), and two species from the subgenus *Petunioides* (*N. langsdorfii and N. sylvestris*). Homologs of gene *mis* were detected in the genomes of *N. glauca, N. tomentosa*, *N. tomentosiformis* and *N. tabacum*, however, the size of the hybridized fragments was different between *N. glauca* and species in the subgenus *Tabacum* and the hybridization pattern in *N. tomentosa* was different from that of the two species in the subgenus *Tabacum* (*N. tabacum* and *N. tomentosiformis*). Since T-DNA fragments of *N. tabacum* were identical to those of *N.tomentosiformis* and were not detected in the genome of *N. sylvestris*, the *mis* gene of *N. tabacum* likely came from *N. tomentosiformis*.

Suzuki et al. (2002) sequenced DNA in *N. glauca* adjacent to the cT-DNA. To investigate regions adjacent to the cT-DNA in other species, Southern hybridization was carried out using either a DNA fragment outside the left or right arms of the cT-DNA of *N. glauca* as a probe. All examined *Nicotiana* genomes showed the presence of sequences homologous to both sides of the cT-DNA suggesting that these are original sequences existing in the genomes of *Nicotiana* plants. Similar size fragments were found in most species of the subgenus *Rustica* and *Tabacum*, which likely represent subgenus-specific restriction fragments. Interestingly, the signals using NgL and NgR as probes fell into the same fragment in the genomes of *N. tomentosa*, *N. tomentosiformis*, *N. tabacum*. Although the same DNA sequences bordering the cT-DNA in *N. glauca* were found in the genome of these three species, the sequences were not contiguous to the cT-DNA therefore the location of the cT-DNA in *N. glauca* is different from that in the species of the subgenus *Tabacum*.

**Figure 2:**
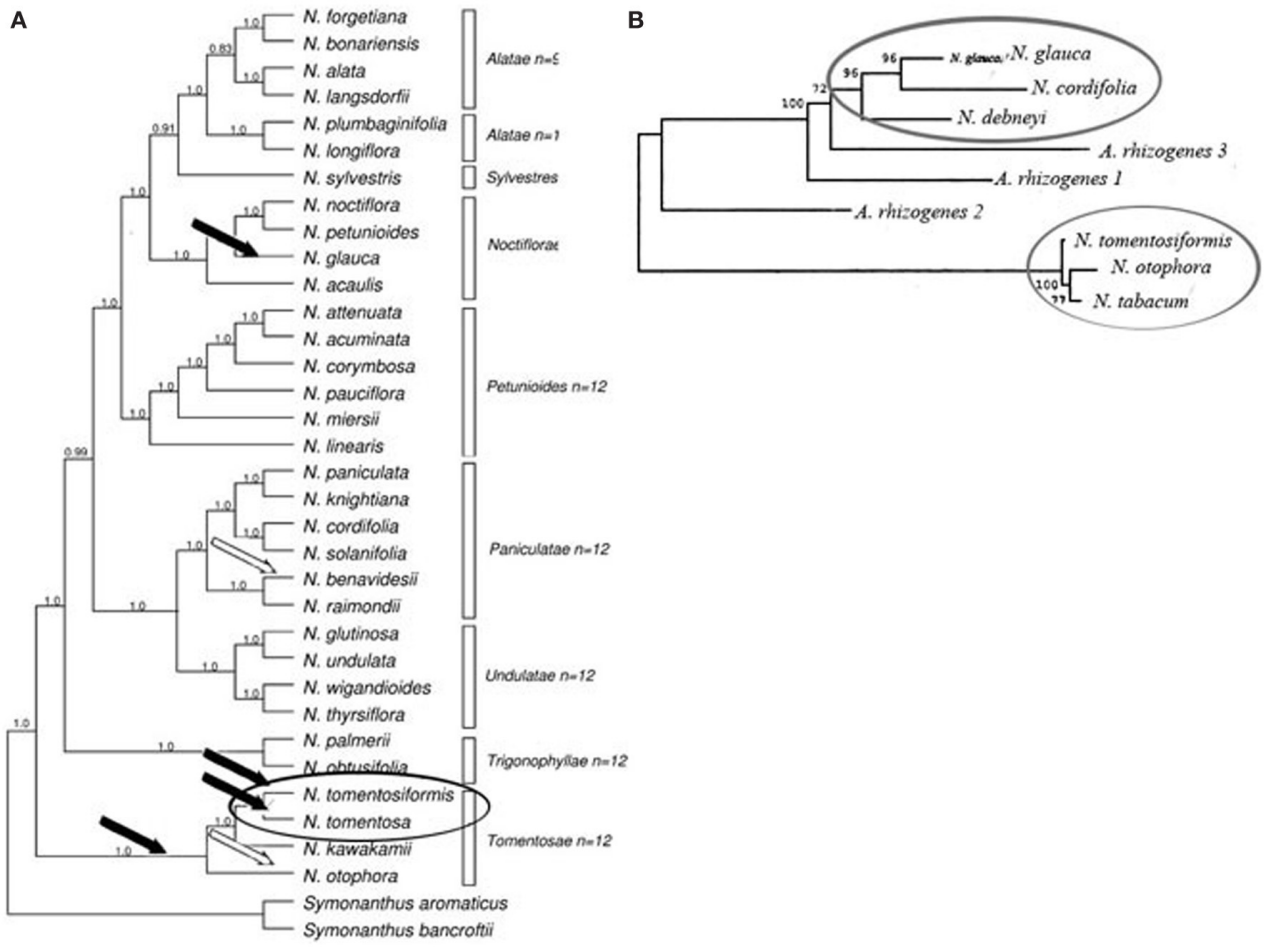
**Phylogenetic analysis of *Nicotiana* (Clarkson et al., 2004), using ITS and chloroplast sequences together with T-DNA marker**. **(A)** Bayesian analysis of diploids only combined dataset (plastid and ITS). Consensus of 40,001 trees with posterior probabilities shown above branches. Bars indicate *Nicotiana* sections according to Knapp et al. (2004), Black and white arrows indicate the deduced insertion events by mikimopine-type or unknown opine-type pRi T-DNAs correspondingly (Suzuki et al., 2002). **(B)** Phylogenetic analysis of *rolC*, gene (Intrieri and Buiatti, 2001) by neighbor-joining method. Ovals show results of possible independent transformation events.

Thus, the phylogenetic analysis undertaken by Intrieri and Buiatti (2001), and the study of opine genes and T-DNA integration sites performed Suzuki et al. (2002), suggest that there have been no less than two acts of *Agrobacterium* mediated transformation in the evolution of *Nicotiana* species (Figure 2). While the comparison of DNA sequences and the detection of *mis* homologs clearly demonstrates that the origin of the cT-DNA in *N. glauca*, *N. tabacum*, *N. tomentosa*, and *N. tomentosiformis* is derived from a mikimopine-type Ri plasmid similar to pRi1724, the origins of the cT-DNA in other species are still unknown.

## Expression of *Nicotiana* cT-DNA genes

pRi transgenic plants exhibit a specific phenotype (dwarfing, loss of apical dominance, increased root mass, and decreased rate of fertilization) (Tepfer, 1984). However, *Nicotiana* species that contain cT-DNA in their genomes show no such phenotype. Are these genes expressed and functional, or they are pseudogenes?

Early experiments by Taylor et al. (1985) did not detect transcripts of the cT-DNA genes in *Nicotiana glauca*. Other researchers were able to detect transcripts of Ng*rolB*, Ng*rolC*, NgORF13, NgORF14 in callus tissues of *N. glauca* (Aoki and Syono, 1999a) as well as in genetic tumors of F1 *N. glauca* × *N. langsdorffii* hybrids, but not in leaf tissues of the same hybrid (Ichikawa et al., 1990; Aoki et al., 1994). Northern analyses of *N. tabacum* oncogenes showed that a *trolC* transcript accumulated in shoot tips and young leaves. The expression pattern of tORF13 was similar to t*rolC*, however, tORF13 expression was detected in flowers (Meyer et al., 1995; Frundt et al., 1998).

Intrieri and Buiatti studied transcription of the cT-DNA genes using RT-PCR in a number of species, using *N. langsdorffii* as a negative control. For their analyses authors used leaves from young *in vitro*-grown plantlets and hormone autotrophic (habituated) callus tissues grown on a hormone-free cultural medium as previously described by Bogani et al. (1985).

In this study *rolB* transcripts were found to be present in all cases in habituated callus tissues, but not in leaves; *rol*C was expressed in calli as well as in leaves of *Rustica* species (*N. glauca* and *N. cordifolia*), and only in calli in species representing the subgenus *Petunioides*; no expression was demonstrated to occur in species from the subgenus *Tabacum*. ORF13 and 14 mRNA was always detected in calli and ORF13 transcripts were found in leaf tissues of *N. tabacum* and *N. tomentosiformis*.

Aoki and Syono (1999b, 2000) analyzed the function of Ng*rol* genes by transforming leaf explants of *N. tabacum* and *N. debneyi* with *A. tumefaciens* that harbored either a *rolB-rolC-*ORF13-ORF14 fragment from pRi or cT-DNA of *N. glauca*. Nearly all of the leaf segments inoculated with pRi fragment developed hairy roots. No significant root growth, however, appeared on the explants treated with *A. tumefaciens* that harbored Ng cT-DNA.

A comparison of the nucleotide sequences of Ng*rolB* and Ri*rolB* indicates that these oncogenes have different length coding regions. Each ORF starts at the same position, but Ng*rolB* ends at early termination codon 633 bp from the initiation site. The authors suggested that *N. glauca* plants do not exhibit the hairy-root phenotype due to the truncation of the Ng*rolB* reading frame.

A comparison of the DNA sequences of Ng*rolC* and Ri*rolC* indicates that the reading frame of Ng*rolC* begins and terminates at the same positions as Ri*rolC. Nicotiana tabacum* leaf disks were transformed with the P35S-Ng*rolC* chimeric construction, yielded transformants that expressed a dramatically dwarfed phenotype, probably because of the reduced length of internodes. The leaves of these P35S-Ng*rolC* transgenic plants were lanceolated and pale green, with floral organs that were thin and small. These characteristics were identical to the phenotype of the P35S-Ri*rolC* transgenic plants, described earlier (Schmülling et al., 1988). Transgene expression was detected only in transformants that demonstrated these characteristic morphological shifts, no transcripts were detected in leaf tissues from a comparable T0 plant demonstrating a normal phenotype (Aoki and Syono, 1999b).

To compare the expression patterns between the Ng*rol* genes of N. glauca and the Ri*rol* genes of Agrobacterium rhizogenes, Nagata et al. (1996) carried out fluorometric and histochemical analyses of the tissues from transgenic genetic tumors, growing on the hybrid of Nicotiana glauca × N. langsdorffii (F1) that contained a beta-glucuronidase (GUS) reporter gene fused to the promoter of (Ng*rol*B, Ng*rol*C, Ri*rol*B, or Ri*rol*C. In all constructs they studied, significantly higher GUS activity was found in tumors than in the other organs (roots, stems, and leaves) of transgenic plants. The tendency toward higher GUS activities in tumors than in normal tissues seen with the Ri*rol*B and Ri*rol*C promoters was also seen with the Ng*rol*B and Ng*rol*C promoters. GUS activities from the rolB promoter expressed in normal F1 plants were, however, different from those seen from the rolC promoter. The expression of the Ri*rol*B and Ng*rol*B promoters in stems, roots, and leaves were 10–100 fold lower than in genetic tumors. Almost no activity was detected in leaves. By contrast, expression from the Ri*rol*C and Ng*rol*C promoters was only 1.5–10 fold lower than in genetic tumors and a significant activity was detected in leaves.

Histochemical analysis of transgenic normal Fl plant tissues showed that Ng*rolB*-GUS and Ng*rolC*-GUS, as well as Ri*rolB*-GUS and Ri*rol*C-GUS, had common tissue-specific expression patterns. Ng*rolB*-GUS normal Fl transgenic plant tissues displayed high GUS activity in the meristematic zones of roots and in the apexes of shoots. A similar pattern of staining was ob-served in the Ri*rolB*-GUS transgenic plants. In the case of Ng*rolC*-GUS and Ri*rolC*-GUS normal Fl transgenic plant tissues, GUS activity was observed primarily in the apices, vascular bundles of leaves, stems, and roots.

Expression of the *mis* gene homologs in *N. glauca* was detected by RT-PCR (Suzuki et al., 2002). It was shown that both homologs of the *mis* gene were amplified by RT-PCR using separate ortholog specific primers. These data support the hypothesis that the *mis* homologs are not pseudogenes. Transgenic plants transformed by the T-DNA of the wild type plasmid Ri1724 or by the *mis* gene alone, synthesize mikimopine in different organs (Suzuki et al., 2001), although no mikimopine accumulation was detected in wild-type *N. glauca* by paper electrophoresis. It is therefore likely that Ng*mis* homologs are transcribed at a very low level.

A full-length Ng*mis*R homolog was isolated and integrated into an expression vector in *Escherichia coli*. The purified Mis protein was able to catalyze synthesis of mikimopine from L-histidine and α-ketoglutaric acid in a reaction buffer supplemented with NADH as a co-factor (Suzuki et al., 2002).

Thus, the oncogenes of *Nicotiana* cT-DNA are expressed in different tissues of present day tobacco plants at a low level and are therefore not pseudogenes.

## cT-DNA and genetic tumors in *Nicotiana*

Genetic tumors appear in certain genotypes spontaneously without being induced by any detectable environmental factor, and the tumor state is hereditary. Spontaneous genetic tumors in *Nicotiana* were first reported by Tanaka (2008). They have been detected throughout the plant and in whole progeny populations of certain crosses of *Nicotiana* species (Kehr and Smith, 1954). In some hybrids, genetic tumors have been reported to be formed irregularly in some of the offspring or limited to certain organs of the plants (Smith, 1958). It has been proposed that certain genes appropriately combined in a hybrid promote the development of these genetic tumors (Naf, 1958; Ahuja, 1968). Naf (1958) divided *Nicotiana* species into two groups, so called “plus” and “minus” groups. The “plus” group consists mainly of the species of the section *Alatae* whereas the minus group contains species from several sections. Crosses between the species within “plus” or “minus” groups do not produce tumorous progeny, while crosses between species from “plus” and “minus” groups do. Ahuja (1968) hypothesized that the species belonging to the “plus” group have a gene or a locus defined as initiator (I) and the species belonging to the “minus” group have a number of genes or loci (ee) for tumor enhancement and expression. For tumor formation both I and ee loci must be present.

Fujita (1994) expect that most species belonging to the minus group contain cT-DNA and that its genes could somehow be associated with the formation of genetic tumors on the *Nicotiana* hybrids. However, since there are no reports showing a connection between the ee genes and cT-DNA genes so far, this promising hypothesis has not yet been validated (Tanaka, 2008). As already mentioned, Ng*rolB*, Ng*rolC*, NgORF13 and NgORF14 genes are transcribed in genetic tumors on *N. glauca* × *N. langsdorffii* F1 hybrids (Ichikawa et al., 1990; Aoki et al., 1994). Some of these genes function in several organs of non-tumorous hybrid plants, like their counterparts in pRi T-DNA (Nagata et al., 1995, 1996; Udagawa et al., 2004). As soon as tumorigenesis is initiated by aging or stress, these genes are active in the developing outgrowth in a regulated manner. This means that a high level of expression of Ng*rol* genes is correlated with tumor formation on an F1 hybrid. However, it has not been determined if the formation of tumors is caused by the expression of Ng*rol* genes (Tanaka, 2008). Moreover, the stem and leaf tissues of *Nicotiana* species accumulate transcripts of the Ng*rol* genes (Meyer et al., 1995; Frundt et al., 1998). These observations suggest that the expression of these Ng*rol* genes might be unrelated to the induction of tumors. Overexpression of Ng*rolC*, NgORF13, or tORF13 cause the proliferation of cells on carrot disks (Frundt et al., 1998), and morphological alterations of tobacco explants, similar to hairy root syndrome on transgenic plants (Aoki and Syono, 1999a,b). Therefore, cT-DNA oncogenes may be responsible for enhancing the development of genetic tumors (Tanaka, 2008).

It is widely discussed that phytohormones contribute to genetic tumor formation. The role of auxin and cytokinins in genetic tumor formation in *Nicotiana*, however, has been disputed. On the one hand, in the light-grown tissues of genetic tumors, indole acetic acid (IAA) was found to be the predominant auxin and its level increased during tumor initiation (Bayer, 1967; Ichikawa et al., 1989). On the other hand, in dark conditions endogeneous IAA remained at a constant, low level throughout the tumorigenetic process (Fujita et al., 1991). A higher cytokinin level was associated with tumorigenesis in tumor-prone hybrid tissues. While analyzing the role of cytokinins in *Nicotiana* genetic tumor formation, Feng et al. (1990) have shown that tumor formation of X-ray-induced non-tumorous mutants of *N. glauca* × *N. langsdorffii* was restored either by the insertion of the *A. tumefaciens ipt* gene, which encodes the key enzyme of cytokinin biosynthesis, or by the addition of cytokinin. Nandi et al. (1990) determined the profile of endogenous cytokinins in genetic tumors of *N. glauca* (Grah.) × *N. langsdorffii* (Weinm.) hybrids. They showed that while zeatin is predicted to be the predominant endogenous cytokinin in tissues of all ages, the genetic tumor tissue derived from this hybrid does not contain notably high endogenous cytokinin levels.

Since tumor growth may be caused not only by high concentrations of hormones, but also by enhanced sensitivity to them, this may explain the contradictory data on the content of hormones in tumors. Even if hormone levels are increased in tumors, it is not necessarily caused by the expression of the cT-DNA genes: *rol* genes can be regulated by hormones. For example, it was shown that expression of Ng*rolB* was induced by auxin, as was Ri*rolB*, probably through the presence of the auxin-responsive *cis*-element ACTTTA found in the promoters of Ng*rolB* and Ri*rolB* which is acted upon by the trans-factor NtBBF1 (Tanaka, 2008).

It is clear that further work will be needed to establish the relationship between cT-DNA oncogenes and genetic tumorigenesis in *Nicotiana*.

## Search for T-DNA-like sequences in other solanaceae species

The Solanaceae is a large angiosperm family containing many economically important crops. *A. rhizogenes* is known to infect species belonging to different Solanaceae genera. Intrieri and Buiatti (2001) attempted to identify T-DNA-like sequences in species belonging to genera *Cestrum*, *Petunia*, and *Solanum* (*C. parqui, C. foetidus, P. hybrida, S. tuberosa, S. melongena, C. annuum*, and *S. lycopersicon*) using the same screening procedure, as they did for *Nicotiana*. None of the species screened showed amplification by PCR and no hybridization was obtained using *A. rhizogenes* and *N. glauca* probes. Kulaeva et al. (2013) extended the analysis to species from genus *Solanum* looking for T-DNA-like sequences. The authors used TaqMan real-time PCR with degenerate primers and probes for *rolB, rolC*, ORF13, ORF14 to analyze the following species: *S. chmielewskii, S. esculentum var. cerasiforme, S*. *glabratum, S. habrochaites, S. peruvianum, S. pimpinellifolium*, *S. cheesmanii, S. parviflorum, S. chilense, S. acaule, S. ajanhuiri, S. albicans, S. andigenum, S. berthaultii, S. boyacense, S. boyacense, S. canarense, S. canarense, S. cardiophyllum, S. chacoense, S. chaucha, S. chocclo, S. curtilobum S. demissum, S. demissum, S. doddsii, S. dulcamara*, *S. fendleri, S. goniocalyx, S. hjertingii, S. hondelmanii, S. hougassi, S. jamesii, S. juzepczukii, S. kurtzianum, S. mamilliferum, S. phureja, S. pinnatisectum, S. pinnatisectum, S. polytrichon, S. riobambense*, *S. rybinii, S. sparsipilum, S. spegazzinii, S. stenotomum, S. stoloniferum, S. tarijense, S. tenuifilamentum, S. tuberosum, S. vernei, S. verrucosum, S. oplocense.* They used *N. tabacum* DNA as positive control and *N. langsdorffii* as negative control. Amplification of specific sequences was not detected in any of the tested species.

This data shows that the presence of cT-DNA is not a feature of whole Solanaceae family, but has only been described to date for members of the genus *Nicotiana*.

## T-DNA in other dicotyledonous families

Given the documented occurrence of cT-DNA, it is reasonable to hypothesize that other plant species, outside of the family Solanaceae, would have been transformed by *Agrobacterium* and contain at least remnants of cT-DNA.

The existence of cT-DNAs in species outside of the family Solanaceae has been reported by several groups. Using Southern analyses, sequences similar to pRi T-DNA were found in the genomic DNA of normal carrot (*Daucus carota*) (Spano et al., 1982), field bindweed (*Convolvulus arvensis*) (Tepfer, 1982); and carpet bugleweed (*Ajuga reptans*) (Tanaka, 2008). These studies did not involve DNA sequencing so they were not able to confirm whether there is T-DNA in the analyzed species.

Prior work in our group (Matveeva et al., 2012) attempted to clarify whether fixed T-DNA is present in other species outside the genus *Nicotiana*, and to evaluate the evolutionary relevance of natural T-DNA transfer. We sought to quickly screen a large number of plant genomes for the presence of T-DNA from both *A. tumefaciens* and *A. rhizogenes* using a modification of TaqMan-based real-time PCR (Livak et al., 1995) that combines the positive features of PCR and DNA blot hybridization in a single reaction (Matveeva et al., 2006). The search was limited to dicotyledonous plants native to temperate zones (mild winter, warm summer, and sufficient rainfall) due to the common occurrence of *Agrobacterium* in soils under these climate conditions.

This work analyzed 127 dicotyledonous plant species belonging to 38 different families. Species included carrot (*Daucus carota*) and field bindweed (*Convolvulus arvensis)*, mentioned above. Each of the plants was screened for DNA sequences homologous to two different sets of T-DNA oncogenes: The first set included sequences homologous to *A. rhizogenes* oncogenes (*rolB, rolC*, ORF13, and ORF14), and the second contained sequences homologous to *A. tumefaciens* oncogenes (*tms1* and *tmr*). Plant DNA samples from 126 species did not display detectable fluorescent signals for the T-DNA genes from either *A. rhizogenes* or *A. tumefaciens*. However, DNA samples isolated from several plants of *L. vulgaris* gave a positive result. In contrast, amplification was not observed using primers for the *tms1* and *tmr* genes of *A. tumefaciens* (Matveeva et al., 2012).

The absence of T-DNA homologs in most of the plant species investigated leads us to the conclusion that HGT from *Agrobacterium* is a rare event in the plants. However, finding cT-DNA sequences in the genomes of species other plants than *Nicotiana* indicates that HGT from *Agrobacterium* to plants occurs outside of this genus.

It is interesting to note that the only examples of HGT demonstrated thus far occur in plants transformed by *A. rhizogenes*, but not *A. tumefaciens.* This may suggest that infection induced by *A. rhizogenes* is more efficient than that induced by *A. tumefaciens* (Tepfer, 1984).

## cT-DNA in the genomes of the genus *Linaria*

Sequences homologous to T-DNA oncogenes in *Linaria vulgaris*, were identified by real time PCR and named Lv*rolB*, Lv*rolC*, LvORF13, LvORF14. BLAST analyses demonstrated the highest level of sequence identity (93%) between Lv*rolC* and Ri*rolC* from the pRiA4 of *A. rhizogenes.* LvORF14 had the lowest similarity to the corresponding *Agrobacterium* oncogene (85%). A homolog of a gene for mikimopine synthase (*mis*) was also identified. To define the full extent of the cT-DNA integrated into *Linaria vulgaris* genome, a chromosome walking approach was performed to identify the upstream fragment of the Lv cT-DNA. This work indicated that the *L. vulgaris* genome contains two copies of cT-DNA which are organized as an imperfect direct repeat. Analysis of the cT-DNA copies demonstrated that both of them contain sequences similar to the following genes: ORF2, ORF3, ORF8, *rolA, rolB*, ORF 13, ORF1, and *mis*. The left side of the repeat contains additional sequence, homologous to part of the agrocinopine synthase (*acs*) gene. Analysis of the flanking regions of the Lv T-DNA was performed by real-time thermal asymmetrical interlaced (TAIL)-PCR with primers and probes to the Lv*mis* gene. The flanking plant DNA identified in this analysis was found to be similar to the Ty3/gypsy-like retrotransposon (Matveeva et al., 2012).

Samples of *L. vulgaris* were collected in the European part of Russia in Moscow, Voronezh and Krasnodar regions, and in the Asian part of Russia in the Novosibirsk, Tumen, and Chelyabinsk regions. The distance between the most western to the most eastern points was about 4000 km. The distance from the most northern to the most southern points was about 2000 km. Two to three plants were analyzed from each of these collection points. All of the samples contained T-DNA-like sequences, however, there was polymorphism among their nucleotide sequences (Matveeva et al., 2012).

Analysis of the sequences for both *L. vulgaris* homologs of *rolC* demonstrated that they contained intact open reading frames. The fragments corresponding to the coding regions of genes Lv*rolB*, LvORF13, LvORF14, and Lv*mis* contain several stop codons or frameshifts that alter the ORFs. An analysis of the expression of these genes was carried out in tissues from the internodes, leaves, and roots of 1 month old, *in vitro* aseptically grown plants using RT real-time PCR. No mRNA corresponding to Lv*rolB*, Lv*rolC*, LvORF13, LvORF14 and Lv*mis* genes was amplifiable from these samples, therefore, the *rol* genes do not appear to be transcribed in *L. vulgaris* (Matveeva et al., 2012).

Toadflaxes (*Linaria* Mill.) form the largest genus within the tribe *Antirrhineae*. *Linaria* includes about 150 species that are widely spread in the Palearctic region, but the representatives of the genus are the most variable in the Mediterranean basin. The origin of the genus has been placed in the Miocene era (Fernandez-Mazuecos and Vargas, 2011) predating the Messinian Salinity Crisis (Hsu et al., 1977). The latest classification of the genus *Linaria* accepts seven sections (*Linaria, Speciosae, Diffusae, Supinae, Pelisserianae, Versicolores, and Macrocentrum*) (Sutton, 1988).

Studies indicate that cT-DNA exists in a number of *Linaria* species belonging to the sections *Linaria* and *Speciosae* (Matveeva and Kosachev, 2013; Pavlova et al., 2013). No cT-DNA was detected in *Linaria* species outside these sections. It appears that *rolC* is concerved among studied *Linaria* species based on the sequencing analysis of *rolC* homologs in *L. genistifolia* subsp. *dalmatica* (sec. *Speciosae*) and *L. acutiloba* (sec. *Linaria*) (Matveeva and Kosachev, 2013; Pavlova et al., 2013).

Thus, HGT of T-DNA from *Agrobacterium* to plants is not limited to *Nicotiana* spp, it has also occurred in the genus *Linaria*. The *rolC* homolog is the most conserved gene among the cT-DNA genes in *Linaria* and *Nicotiana* spp. In both genera plants were transformed by a mikimopine strain of *A. rhizogenes.*

## Possible function of T-DNA in plant genomes

The existence of several independent acts of *Agrobacterium* mediated transformation of plants and the maintenance of the cT-DNA in plant genomes during the process of evolution propose, that T-DNA-like sequences may give some selective advantages to the transformed plants (Ichikawa et al., 1990; Matveeva et al., 2012).

Suzuki et al. (2002) mentioned two possible functions of cT-DNA: increasing root mass leading to tolerance to drought, and changing the biological environment, particularly the soil microbiome represented by root-associated bacterial populations. Increasing root mass would seem beneficial for tolerance to dry conditions. Hence, ancient transformed plants with increased root mass might have demonstrated increased tolerance to dry environments surviving in arid conditions (Tanaka, 2008). However, no phenotype of the hairy root disease is observed in *Nicotiana* and *L. vulgaris* plants. In contrast, *L. vulgaris* explants show *in vitro* a shooty phenotype and in representatives of both genera *rolB* is mutated. Among the oncogenes of pRi T-DNA, *rolB* gene function seems to be the most important for hairy root induction because transformation of plants by the Ri*rolB* gene alone can induce hairy root formation. In contrast to the pRi*rolB*, the Ng*rolB* gene alone or in combination with other *N. glauca* homologs of *A. rhizogenes* oncogenes did not induce adventitious roots (Aoki and Syono, 1999a,b).

Aoki and Syono (1999b) performed base substitutions at two nucleotide positions, using site-directed mutagenesis, with the aim of producing a full-length form of Ng*rol*B capable of stimulating adventitious root induction. Transgenic plants overexpressing this altered Ng*rolB* demonstrated typical morphogenetic abnormalities. This experiment shows the possibility that a functional *rolB* gene may have operated during early steps of the evolution of transgenic *Nicotiana*.

Identification and sequencing of the *mis* homologs in *Nicotiana* and *Linaria* suggests that the origin of their cT-DNA is probably the mikimopine Ri plasmid. The presence of this gene may be related to plant–microbe interactions. Oger et al. (1997, 2000) reported that producing opines in genetically modified plants alters their ecological environment, in particular, changing the soil microbiome and root-associated microbe populations. If the synthesis of opines were beneficial for a plant species (even at a low level, in a specific tissue, or at a specific stage of oncogenesis), it may impact the appearance of advantageous plant–bacterium interactions. Plants maintaining cT-DNA in the genome could potentially maintain certain species of microorganisms in their rhizosphere via the secretion of opines in the root zone. Such potentially beneficial bacteria in the rhizosphere may in turn influence the root microbiome and convey nutritional and/or defensive features.

Early flowering or a shift from biennial to annual lifecycle without vernalization can take place on pRi transgenic *Cichorium intybus* and *Daucus carota* plants (Limami et al., 1998). These flowering features are beneficial when propagating such transgenic plants over the untransformed parentals. When considering the adaptational potential of natural transformation, the authors focused on the occurrence of flowering in the absence of a cold treatment. Given the mobility of seeds by wind, animals, and water, it is likely that biennial varieties or ecotypes may be transported to the southern latitudes where annualism would be beneficial. However, *Nicotiana* and *Linaria* species are not biennials (Goodspeed, 1947; Sutton, 1988; Blanco-Pastor et al., 2012). In addition, cT-DNA containing *Linaria* species from the sections *Linaria* and *Speciosae* are perennial, while other sections contain annual species. It is interesting to note that the cT-DNA containing sections are found worldwide while other sections are in the Mediterranean region and the Pyrenees (Table 2) (Sutton, 1988). It is unclear if this observation is due to the rarity by which plants acquire permanent cT-DNA, or if its foundation is related to some fitness benefit conferred by the cT-DNA. It can be speculated, however, that the ecological plasticity of species within the sections *Linaria* and *Speciosae* is somehow associated with the presence of cT-DNA in their genomes.

**Table 2:** **Major features of infrageneric taxa of the genus *Linaria* (according to Sutton, 1988)**.

**Section**	**Habit**	**Distribution**
*Linaria*	Perennial	Eurasia
*Speciosae*	Perennial	Europe
*Diffusae*	Annual or perennial	Mediterranean
*Supinae*	Annual or perennial	Mediterranean
*Pelisserinae*	Annual or perennial	Mediterranean
*Versicolores*	Annual or perennial	Mediterranean, Iberian Peninsula
*Macrocentrum*	Annual	Mediterranean

It would appear, therefore, that annualism is not related to natural transformation in *Nicotiana* and *Linaria.*

It is interesting to note that *rolC* is the most conserved gene among the cT-DNA oncogenes found in *Nicotiana* and *Linaria* (Intrieri and Buiatti, 2001; Mohajjel-Shoja et al., 2011). In some representatives of the *Nicotiana*, only *rolC* is able to encode a functional product (Mohajjel-Shoja et al., 2011). The same trend was observed for *Linaria* T-DNA-like sequences (Matveeva et al., 2012; Matveeva and Kosachev, 2013). The function of *rolC*, however, is poorly understood. It has been speculated that the product of *rolC* releases cytokinins from conjugates (Estruch et al., 1991). Other researchers demonstrated that the RolC protein participates in the processes of sucrose metabolism and/or transport (Nilsson and Olsson, 1997; Mohajjel-Shoja et al., 2011). RolC has also been proposed to promote somatic embryogenesis in plants (Gorpenchenko et al., 2006). Such data are consistent with a cytokinin function of the gene. Constitutive expression of *rolC* in cultured plant tissues activates secondary metabolism: the *rolC* gene alone increases production of tropane alkaloids, pyridine alkaloids, ginsenosides, and anthraquinones among others (Bulgakov et al., 1998, 2002; Palazon et al., 1998a; Bonhomme et al., 2000a,b; Bulgakov, 2008) and stimulates the expression of pathogenesis-related proteins (Kiselev et al., 2007). It is unclear how the *rolC* gene product mediates such pleiotropic effects, further biochemical characterization of RolC is required. This is complicated by the fact that *rolC* has no significant homology with any other genes (of prokaryotic or eukaryotic organisms) whose function is known (Bulgakov, 2008).

Activation of secondary metabolism in transformed cells may be due to the action of other *rol* genes (Chandra, 2012). Shkryl et al. (2008) studied the influence of *rol* genes products on secondary metabolism of *Rubia cordifolia.* They investigated *rol* genes individually and studied their combined action. They found that individual *rolA, rolB*, and *rolC* genes were able to stimulate biosynthesis of anthraquinones in transformed calli. The strongest anthraquinone—stimulating activity was detected for an *R. cordifolia* culture overproducing RolB where they saw a 15-fold increase of anthraquinone accumulation as compared to untransformed calli. The *rolA*- and *rolC*-expressing calli produced 2.8- and 4.3-fold higher amounts of anthraquinones, correspondingly. Palazon et al. (1998b) reported that the *rolA* gene stimulated production of nicotine.

Thus, increasing the amount of secondary metabolites is a characteristic of tissues where *rol* genes are expressed. This property can be useful for plants, because secondary metabolites may contribute to the resistance of plants to pests. It seems likely that a possible function cT-DNA is to mediate how plants interact with their environment by secreting opines and/or by changing the amounts of secondary metabolites. It will be essential to confirm such hypotheses through additional experimentation that might include silencing or excision experiments that are now possible using CRISPR technology (Qi et al., 2013).

The study of the long term impacts of HGT by *Agrobacterium* in plant lineages is in the early stages. However, we can note some trends:

– HGT of T-DNA from *Agrobacterium* to plants occurred in the evolution of several genera, at least *Nicotiana* (family Solanaceae) and *Linaria* (family Plantaginaceae);
– in both genera plants were transformed by a mikimopine strain of *A. rhizogenes*;
– a *rolC* homologe is the most conserved gene among the T-DNA genes in *Linaria* and *Nicotiana* spp;
– In *Linaria vulgaris* and *Nicotiana glauca* there are more than one copy of T-DNA per genome.

Continued studies of the genetic and biochemical effects of cT-DNA integration in naturally transgenic plants are important and will continue to provide insights into the impact of such rare acquisitions on plant evolution.

### Conflict of interest statement

The authors declare that the research was conducted in the absence of any commercial or financial relationships that could be construed as a potential conflict of interest.
